# The Caribbean under the radar? A scoping review of carbapenemase-producing Enterobacterales across the region

**DOI:** 10.1371/journal.pgph.0007176

**Published:** 2026-08-26

**Authors:** Saied Ali, Ansara Baig, Rajeev Peeyush Nagassar, Liam P. Burke, Fidelma Fitzpatrick, Deirdre Fitzgerald-Hughes

**Affiliations:** 1 Department of Clinical Microbiology, Royal College of Surgeons in Ireland, University of Medicine and Health Sciences, Dublin, Ireland; 2 Department of Clinical Microbiology, Beaumont Hospital, Dublin, Ireland; 3 Department of Medicine, Point Fortin Hospital, South West Regional Health Authority, Point Fortin, Trinidad and Tobago; 4 Department of Microbiology, Sangre Grande Hospital, Eastern Regional Health Authority, Sangre Grande, Trinidad and Tobago; 5 Antimicrobial Resistance and Microbial Ecology Group, School of Medicine, University of Galway, Galway, Ireland; Washington University School of Medicine, UNITED STATES OF AMERICA

## Abstract

Carbapenemase-producing Enterobacterales (CPE) are a critical antimicrobial resistance (AMR) threat globally. The Caribbean region, despite its high global connectivity, varied healthcare infrastructure and rising antimicrobial use, remains underrepresented in CPE surveillance data. Our study aims to map and characterise the existing literature on CPE in the Caribbean, including bacterial species, carbapenemase types, detection methods, and epidemiological trends. A scoping review was conducted in accordance with PRISMA-ScR guidelines. MEDLINE, EMBASE, Cochrane CENTRAL and Web of Science were searched to 1^st^ July 2025 in addition to grey literature (policy documents, guidelines, websites). Studies were included if they reported carbapenemase production among Enterobacterales from human, animal, or environmental sources in Caribbean nations (defined geophysically). Non-Caribbean studies and non-Enterobacterales organisms were excluded. Fourteen studies published between 2008 and 2024 met inclusion criteria. Reports originated from six countries: Cuba, Puerto Rico, Jamaica, Guadeloupe, the Dominican Republic, and Curaçao. *Klebsiella pneumoniae* was the most frequently reported species. The most common carbapenemase enzymes were KPC (including KPC-2 and KPC-8), NDM-1, NDM-5 and OXA-48. Co-production of NDM and KPC was documented. Detection was largely hospital-based, though community and environmental isolates were also identified. High levels of AMR were observed, with colistin often the only active agent. Surveillance gaps are evident across many populous Caribbean nations. CPE are established in multiple Caribbean countries, but surveillance remains irregular and limited. Addressing critical gaps in diagnostic capacity, reporting and molecular surveillance is essential to ensure the region’s inclusion in the global AMR response.

## Introduction

Antimicrobial resistance (AMR) remains one of the most pressing global public health threats. Carbapenemase-producing Enterobacterales (CPE) have emerged over the past two decades as a major subset of multidrug-resistant organisms (MDROs) characterised by resistance to last-resort antimicrobials, namely carbapenems, their high transmission potential and association with poor clinical outcomes [1,2]. Their global spread has been facilitated by international travel and migration, medical tourism, trade and deficiencies in infection prevention and control (IPC), reinforcing the transboundary nature of AMR [3].

Described in the early 1990s with the identification of KPC-1 in the United States of America (USA), CPE remained a relatively sporadic phenomenon until the early 2000s, when clonal expansion of KPC-producing *Klebsiella pneumoniae* (notably ST258) in the USA and parts of Europe [4,5], followed by the global emergence of NDM-1 from the Indian subcontinent [6], signalled a new phase in the international AMR crisis. By the mid-2010s, OXA-48 and OXA-48-like enzymes had spread across the Mediterranean basin and IMP and VIM types were increasingly detected in Southern Europe, Latin America and Asia [7–9]. The threat posed by CPE was formally recognised by the World Health Organization (WHO) in 2017, when carbapenem-resistant Enterobacterales (CRE) were included in the highest priority tier of its global priority pathogen list, underscoring the urgent need for new antimicrobials and surveillance strategies [10].

In response, international surveillance systems have expanded significantly over the past decade. The WHO’s Global Antimicrobial Resistance and Use Surveillance System (GLASS), launched in 2015, has grown to include over 100 reporting countries spanning all six WHO regions, though data completeness and laboratory capacity remain uneven [11]. In the European Union, the European Antimicrobial Resistance Surveillance Network (EARS-Net) has provided robust, population-level data from 30 countries of the AMR profile of selected invasive isolates, showing that over two-thirds of participating countries now report carbapenem-resistant *K. pneumoniae* bloodstream infections (BSIs), with the highest prevalence in Southern and Eastern Europe [12]. Although carbapenem resistance in *E. coli* remains less common, its increasing incidence, especially among international high-risk clones such as ST131 and ST410, has raised concern for broader dissemination [13,14]. The US Centers for Disease Control and Prevention’s Antibiotic Resistance Laboratory Network (AR Lab Network) and similar regional platforms in Canada, Australia and parts of Asia-Pacific have also contributed to global early-warning efforts [15–17].

Nonetheless, critical surveillance gaps persist in many low- and middle-income countries (LMICs) and small island developing states (SIDS), particularly in sub-Saharan Africa, parts of Southeast Asia and the Caribbean, where laboratory infrastructure, diagnostic capacity and routine AMR reporting remain limited [18,19]. In the 2025 WHO GLASS report, only 27 of 47 countries in the WHO African Region and 7 of 35 countries in the Pan American Health Organization (PAHO) Region, which includes the Caribbean, contributed AMR data. Trinidad and Tobago was the only Caribbean country to submit data to GLASS [11,20]. Furthermore, among reporting countries, substantial variation exists in the number of surveillance sites, specimen types collected and the range of pathogens reported, leading to under-representation of local epidemiological trends and delayed detection of emerging AMR threats [11,20–22].

The Caribbean represents a uniquely vulnerable yet under-researched region. Characterised by diverse healthcare infrastructures, porous inter-island travel routes, high burdens of communicable diseases and rising antimicrobial consumption, the Caribbean may serve as both a reservoir and transit point for AMR [23].

On 26^th^ September 2024, at the United Nations’ General Assembly’s 79^th^ session, world leaders adopted a political declaration committing to reduce AMR-related deaths by 10% by 2030 and to mobilize at least US$100 million toward bolstering surveillance, stewardship and laboratory capacity [24,25]. Prime Minister, Mia Mottley, of Barbados, Chair of the Global Leaders Group on AMR, powerfully framed AMR as a “silent, slow‑motion pandemic” disproportionately impacting LMICs and SIDS. She urged immediate investment in microbiology laboratories, genomic surveillance, and IPC, including rapid diagnostic systems, to ensure timely detection of resistant pathogens and effective response measures. Her broader call for a “reset” in global priorities emphasized that SIDS deserve a seat “at the table” to set their own development agendas and strengthen institutional resilience, including in health systems [26,27].

Studying the epidemiology of CPE in the Caribbean is therefore not only essential to strengthen local antimicrobial stewardship (AMS) and IPC programmes, but also critical to inform global AMR containment strategies. This scoping review aims to thematically synthesise and map the available literature on the presence of CPE in the Caribbean, with a focus on describing country-specific reports, bacterial species, carbapenemase types and detection methodologies. By identifying current gaps in surveillance and research, this review seeks to support regional policy development and promote the Caribbean’s inclusion in the global AMR response.

## Methods

This scoping review was conducted in accordance with the methodological framework outlined in the Preferred Reporting Items for Systematic Reviews and Meta-Analyses extension for Scoping Reviews (PRISMA-ScR) [28–30]. The aim was to answer the research question: “What is known from the published literature about CPE in the Caribbean, specifically in relation to bacterial species, carbapenemase types, detection methods and epidemiological patterns?”

Given the fragmented and limited nature of surveillance in the region, a scoping review approach was selected to provide a comprehensive overview of existing evidence and to highlight critical knowledge gaps to inform future research and policy development.

The bibliographic databases; MEDLINE (via PubMed), EMBASE, Cochrane CENTRAL and Web of Science were searched from inception to 1^st^ July 2025, using a combination of keywords and Boolean operators (see S1 Appendix). In line with scoping review methodologies, the search was supplemented with relevant non-peer reviewed relevant grey literature (e.g., policy documents, reports, guidelines). Bibliographies of included articles were systematically reviewed to identify additional potentially eligible studies.

For the purposes of this review, a Caribbean country was defined as one situated on the Caribbean tectonic plate, in alignment with a geophysical rather than purely political or linguistic classification. This definition encompasses 20 countries and territories, including island states and selected mainland regions located on the Caribbean plate. As such, inclusion may differ from conventional geopolitical definitions of the Caribbean, particularly for mainland territories that are geographically part of continental landmasses but lie on the Caribbean tectonic plate. The full list of included countries and territories is provided in the Search Strategy section of the S1 Appendix.

Studies were eligible for inclusion if they reported the presence of CPE in human, animal or environmental sources within the Caribbean. Primary studies conducted in English or Spanish that documented the detection of Enterobacterales harbouring carbapenemase genes were included. In addition, to align with the objective of mapping the available evidence where context-specific gaps existed (i.e., fragmented research, under-resourcing in the region), systematic reviews, narrative reviews and case reports/series were included in the search. These were included as sources of secondary data (systematic reviews) or supplemental evidence to provide contextual insights (narrative reviews) or to highlight the trajectory of emerging CPE strains in the region (case reports). English and Spanish languages were selected to align with both the dominant languages used in the region and the reviewers’ capacity for accurate screening and data extraction. Studies reporting relevant Enterobacterales spp. in addition to non-Enterobacterales spp (e.g., *Acinetobacter baumannii* or *Pseudomonas aeruginosa*) were eligible. However, in these cases, only those organisms within the scope (CPE) were part of the analysis. Studies that focused solely on non-Enterobacterales organisms were excluded, as were those conducted outside the Caribbean, even if CPE was described.

Titles and abstracts were screened for relevance by two reviewers, SA and AB, followed by full-text evaluation to determine eligibility based on the predefined inclusion and exclusion criteria. Discrepancies or uncertainties were resolved through discussion and consensus.

A descriptive thematic analysis was applied to characterise and map the CPE epidemiology data from the identified studies. Variables extracted included country of study, year(s) of data collection, bacterial species, identified carbapenemase gene(s) or enzyme(s), antimicrobial susceptibility profiles, detection methodology (e.g., polymerase chain reaction (PCR), carbapenemase Nordmann-Poirel (Carba NP), whole genome sequencing (WGS)), clinical setting (e.g., intensive care unit (ICU), outpatient) and relevant epidemiological findings, such as outbreak description, risk factors or treatment outcomes.

## Results

**Fig 1:**
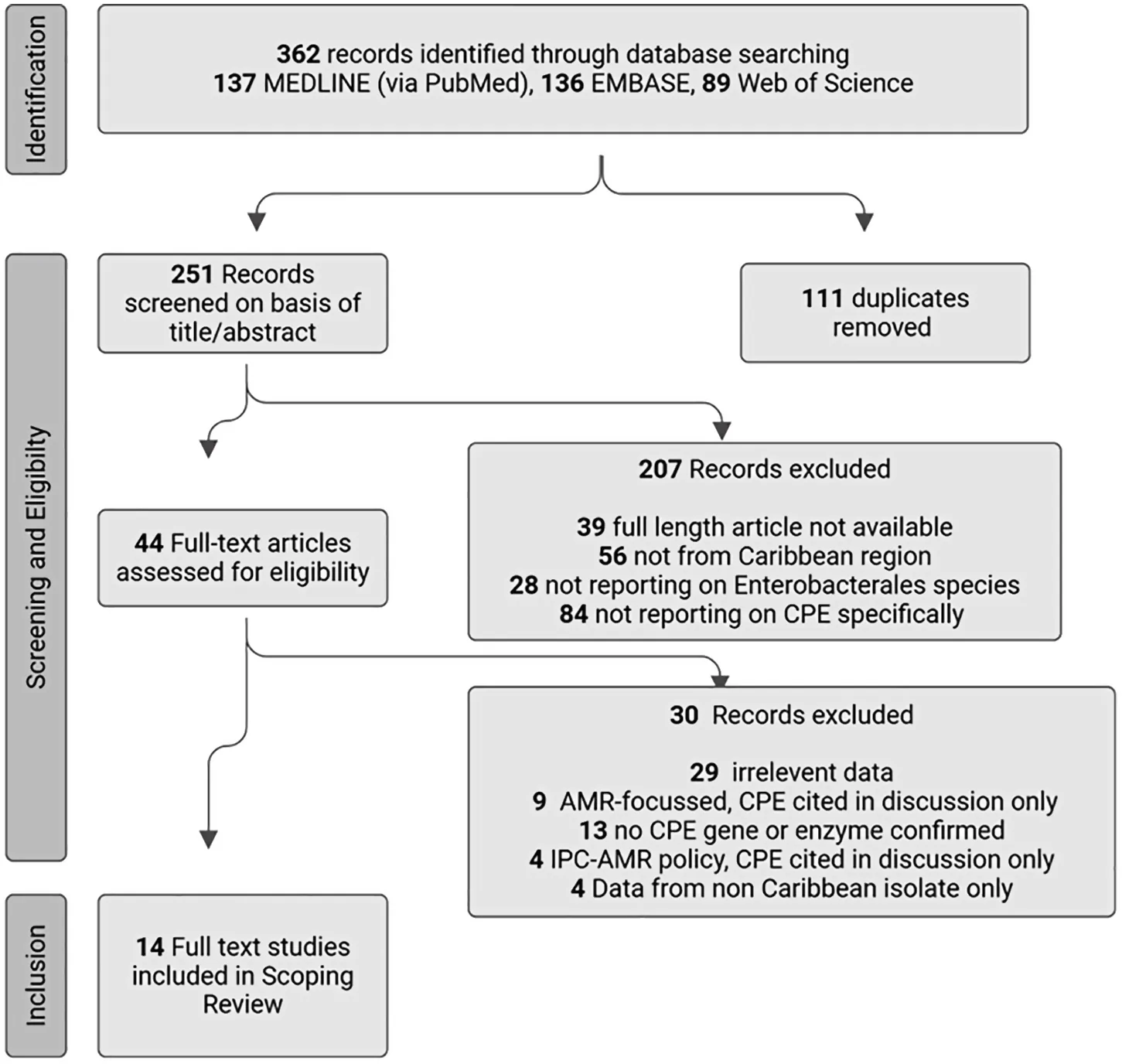
Preferred Reporting Items for Systematic Reviews and Meta-Analyses (PRISMA) flow diagram of search strategy and study selection.

Following a comprehensive literature search and screening process (Fig 1), fourteen studies conducted between 2008 and 2024 met the inclusion criteria. For each study, a summary of the CPE epidemiological data and key observations to support thematic analysis are presented in Table 1 [31–44].

**Table 1:** Characteristics of Included Studies on Carbapenemase-Producing Enterobacterales (CPE) in the Caribbean, 2007–2023 (n = 14).

Author/s, year Reference	Study details		CPE Characteristics		Detection & Diagnostic Methods	Key Observations
	Country; Year/s	Study Type	Number of CPE Isolates/Total Tested (source)	Species (n)^α^; CPE Gene/s Antimicrobial Resistance Data		
Quiñones et al. 2014 [31]	Cuba; 2013	National Surveillance	3/Not specified (clinical infection)	*K. pneumoniae* (3); KPC-2 All strains resistant to β-lactams, fluoroquinolones, aminoglycosides; 1 strain was resistant to colistin	AST, MLST, PCR	Local clonal KPC-2 outbreak (ST1271); high mortality (2/3)
Yu et al. 2022 [32]	Cuba; 2016–2021	National surveillance	152/357 (clinical infection)	*K. pneumoniae* (106), *E. coli* (9), *E. cloacae* (20), Other Enterobacterales; NDM-1, NDM-7, KPC High resistance to β-lactams (100%), aminoglycosides (90%), fluoroquinolones (89%), trimethoprim-sulfamethoxazole (96%); 73% susceptible to colistin, 63% to tigecycline	AST, PCR, WGS	Endemic NDM circulation across 11 hospitals; occasional KPC co-production
Yu et al. 2022 [33]	Cuba; 2017–2021;	Observational	124/Not specified (clinical infection)	*K. pneumoniae* (88), *E. coli* (7), *E. cloacae* (16), Other Enterobacterales; NDM-1, KPC 100% resistant to β-lactams and aminoglycosides; 87% resistant to ciprofloxacin; 75% susceptible to colistin	AST, MLST, PCR, plasmid replicon typing	Widespread NDM-1 with multidrug resistance; colistin-based therapy associated with improved outcomes in BSIs
Erkens-Hulshof et al. 2014 [34]	Curaçao; 2010–2011;	Surveillance	7/Not specified (clinical infection ± colonisation)	*K. pneumoniae* (6), *C. freundii* (1); KPC-2, KPC-3 Meropenem MICs 1–32 mg/L	Check-MDR CT101, MLST, PCR, PFGE, VITEK-2	Introduction of KPC via international transfer from the UK and Colombia; clonal spread of ST11 and ST258
Calderón et al. 2020 [35]	Dominican Republic; 2017	Environmental Surveillance	55 environmental isolates (15 sequenced)	*E. coli, E. cloacae, C. freundii;* KPC-3, OXA-72 96.7% resistant to 3rd generation cephalosporins; 70% to carbapenems; 67% to fluoroquinolones, 42% to aminoglycosides	AST, PCR, WGS	Environmental reservoir of CPE (Isabela River); IncFIB, IncFIA and IncHI1B plasmids detected
Bastian et al. 2011 [36]	Guadeloupe; 2014	Case report	1/1 (colonisation)	*K. pneumoniae* (1); NDM-1 Resistant to all β-lactams, trimethoprim-sulfamethoxazole, fluoroquinolones, gentamicin; susceptible to amikacin, tigecycline, colistin; MICs for imipenem was 16 mg/L, meropenem 32 mg/L, ertapenem 32 mg/L and doripenem 12 mg/L	AST, MLST, PCR, PFGE, plasmid replicon typing, WGS	Imported NDM-1 case from Cuba with novel ST; healthcare-associated acquisition
Breurec et al. 2015 [37]	Guadeloupe; 2014	Case report	1/1 (clinical infection)	*E. coli* (1); OXA-48 Resistant to β-lactams, fluoroquinolones, trimethoprim-sulfamethoxazole; susceptible to 3rd generation cephalosporins, imipenem, meropenem, aminoglycosides, colistin, fosfomycin, tigecycline	AST, Carba NP, MLST, PCR, plasmid replicon typing	Sequence type ST224; patient was originally from Dominica; first OXA-48 report in Caribbean
Thoms-Rodriguez et al. 2016 [38]	Jamaica; 2015	Case report	1/1 (colonisation)	*K. pneumoniae* (1); NDM-1 Resistant to all β-lactams, aminoglycosides, fluoroquinolones; susceptible to tigecycline, aztreonam	AST, MLST, PCR	First NDM in Jamaica; IncA/C plasmid with international healthcare exposure in India
Stone et al. 2023 [39]	Jamaica; 2020	Case report	2/64 (clinical infection)	*K. pneumoniae* (2); NDM-5 Resistant to β-lactams, ciprofloxacin, TMP-SMX, carbapenems; susceptible to gentamicin	AST, MLST, PCR, plasmid replicon typing	ST11 NDM-5 outbreak; IncX3-mediated plasmid spread; high mortality
Gregory et al. 2010 [40]	Puerto Rico; 2008	Outbreak report	7/28 (clinical infection ± colonisation)	*K. pneumoniae* (7); KPC-8, KPC-2 Resistant to β-lactams, aminoglycosides; susceptible to tigecycline	AST, PCR, PFGE	ICU outbreak caused by novel KPC-8 variant with clonal spread
Robledo et al. 2011 [41]	Puerto Rico; 2009–2013	Surveillance	394/10,507 (clinical infection ± colonisation)	*K. pneumoniae* (333), *E. coli* (61); *KPC*-2, KPC-3 High resistance to β-lactams, ciprofloxacin, gentamicin, trimethoprim-sulfamethoxazole	AST, PCR, PFGE	Widespread endemic KPC-2 and KPC-3 in clinical isolates
Escandón-Vargas et al. 2017 [42]	Multi-country Caribbean; Pre-2016	Narrative Review	Not specified (review data; clinical infection ± colonisation)	*K. pneumoniae, E. coli, Enterobacter* species, KPC, NDM, VIM, IMP, OXA-48 High resistance to carbapenems and other β-lactams; co-resistance common; variable susceptibility to colistin and novel agents	AST, PCR, WGS	Early regional documentation of KPC, NDM and OXA-48; major surveillance gaps
García-Betancur et al. 2020 [43]	Multi-country Caribbean; 2016–2019	Narrative review	Not specified (review data; clinical infection ± colonisation)	Enterobacterales; KPC, NDM, VIM, IMP, OXA-48, OXA-23, OXA-58 High resistance to carbapenems and other β-lactams; co-resistance common; variable susceptibility to colistin and novel agents	AST, PCR, PFGE, WGS	Multiple carbapenemases co-circulating; diagnostic limitations highlighted
Thomas et al. 2022 [44]	Multi-country Caribbean and Latin America; 2020–2021	Point prevalence study	Not specified (regional and national surveillance; clinical infection ± colonisation)	*K. pneumoniae, E. coli, E. cloacae;* KPC, NDM, OXA-48, VIM, IMP All isolates resistant to carbapenems, cephalosporins; 96% to ciprofloxacin; 93% to gentamicin; 100% susceptible to colistin	Carba NP, PCR, VITEK 2	Marked increase in CPE detection during COVID-19 pandemic

^*a*^ *Numbers omitted from parentheses where not available.*

*AST: Antimicrobial susceptibility testing; MLST: Multilocus sequence typing; PCR: Polymerase chain reaction; PFGE: Pulsed-field gel electrophoresis; WGS: Whole-genome sequencing.*

### Geographic distribution and characteristics of evidence sources

Collectively, these studies provided insights into the epidemiology, microbiological characteristics, and clinical context of CPE across six of the twenty defined Caribbean nations, namely Cuba, Puerto Rico, Jamaica, Guadeloupe, the Dominican Republic, and Curaçao. The studies comprised; single-centre investigations, multicentre surveillance reports (primary data sources) encompassing clinical, environmental, and epidemiological data. The study contexts and designs varied and included, outbreak investigations, surveillance and observational studies. Supplemental data sources included narrative reviews and case reports. No eligible reports were identified from grey literature searches. Studies employed molecular and/or phenotypic methods to detect carbapenemase genes, most commonly using PCR. Additional tools such as VITEK 2 supported phenotypic resistance profiling, while methods like multi-locus sequence typing (MLST), plasmid replicon typing, WGS and Carba NP were employed in select studies for detailed strain, plasmid or phenotypic characterisation.

### Microbiological landscape: Dominant species and carbapenemase types

*Klebsiella pneumoniae* was the most frequently isolated organism, reported in 12 of the 14 included studies (86%) (Table 1) [31–34,36,38–42,44]. Other Enterobacterales detected included *Enterobacter cloacae* complex [32,33,35,42,44], *Escherichia coli* [32,33,35,37,41,42,44], *Klebsiella aerogenes* and *Serratia marcescens*, with occasional identification of *Citrobacter* and *Morganella* species [32–35,43].

With respect to carbapenemase types, the *Klebsiella pneumoniae* carbapenemase (KPC) enzyme was the most frequently reported across the region, reported in 11 of 14 studies (78.6%) [31–35,40–45]. It was particularly prominent in Cuba and Puerto Rico, often in association with healthcare-associated transmission [31,40,41]. Notably, two variants of KPC, KPC-2 and KPC-8, were identified, with the latter linked to a healthcare-associated outbreak in Puerto Rico [40].

In addition to KPC, the NDM enzyme was reported in nine of the 14 included studies (64%), with cases documented across multiple countries, including Cuba, Jamaica, and Guadeloupe [32,33,36,38,39,42–44]. Both NDM-1 and NDM-5 variants were detected, with some isolates demonstrating co-production of NDM and KPC, as described in a recent multicentre study from Havana [32]. These co-producing strains exhibited high levels of resistance to all antimicrobial classes tested, with the exception of colistin. Strains were also found to harbour IncX3, IncFIB(K), and IncR plasmids and were linked to global high-risk clones, ST11 and ST336 [38,39]. Several cases were associated with prior healthcare exposure outside and within the Caribbean region, including India and Cuba [36,38].

The OXA-48 carbapenemase, while less frequently reported than other carbapenemase enzymes (n = 6, 40%), was identified in Guadeloupe, where its presence in an *E. coli* isolate highlighted the risk of inter-regional spread linked to patient transfers and travel [37]. In one instance, an isolate was recovered from a patient in Guadeloupe who had a recent travel history involving Dominica. OXA-48 and OXA-48-like producers were also reported in multi-country Caribbean studies, while environmental samples from the Dominican Republic identified OXA-72, an OXA-24/40-like carbapenemase [35,42–44].

### Identification methods, molecular epidemiology and high-risk clones

PCR-based detection of carbapenemase genes was universally applied across all 14 studies, while phenotypic confirmation using the Carba NP test was reported in only two studies (13.3%) [37,43], and next-generation sequencing was conducted in only three studies (20%) [32,35,36]. MLST analysis, performed in seven studies (46.7%) [31,33,34,36–39], identified international high-risk clones, including ST11 and ST258 among KPC producers [34], and ST11 and ST336 among NDM producers [38,39]. Plasmid-mediated dissemination of resistance was noted in several studies, with plasmid replicon typing conducted in four studies (26.7%), revealing common plasmid types such as IncR, IncA/C, IncX3, and IncHI1B [33,36,37,39].

### Clinical and environmental sources of CPE

CPE were detected in a variety of settings; the majority being healthcare-associated. ICUs and urology wards were prominent sources of isolates [31–33,44], though community-acquired urinary tract infections associated with CPE were also reported in Jamaica [38]. Across studies, isolates were derived from both clinical infection and colonisation, although numerical differentiation between these sources was inconsistently reported. The primary clinical specimens included blood, urine, respiratory secretions, and wound swabs.

Environmental sources of CPE appear understudied in the region. One study identified NDM-1-producing *E. coli* from the Isabela River in Santo Domingo, highlighting a potential environmental reservoir and transmission route. Environmental surveillance further identified plasmids such as IncFIB and IncHI1B among resistant strains [35]. No eligible studies reported CPE from animal sources.

Importantly, a point prevalence study highlighted an increase in CPE detection during the coronavirus disease 2019 (COVID-19) pandemic period [44]. The authors reported an associated increase in empirical use of broad-spectrum antimicrobials, prolonged hospital stays and overburdened IPC systems during the pandemic as contributing to these trends.

### Antimicrobial susceptibility profiles and treatment outcomes

Antimicrobial susceptibility profiles across all included studies demonstrated high levels of resistance to all β-lactam classes, including third-generation cephalosporins and carbapenems, with resistance to carbapenems often exceeding 90%. Colistin remained one of the few agents that retained meaningful activity against CPE, although reported susceptibility varied between studies; with approximately one-quarter of isolates demonstrated reduced susceptibility [31–33,42,43]. Resistance to aminoglycosides, fosfomycin, tigecycline and trimethoprim-sulfamethoxazole was frequently observed, further limiting therapeutic options. A Cuban study reported universal resistance to β-lactams and aminoglycosides among CPE isolates, with susceptibility preserved in 73% for colistin and 63% for tigecycline [32]. While colistin-based combination therapy was linked to improved outcomes in BSIs, mortality remained ≥50%, particularly in patients with pneumonia or sepsis [33].

### Epidemiological risk factors for infection

Several studies examined epidemiological risk factors associated with CPE infection. Prior use of carbapenems, broad-spectrum cephalosporins and multiple antimicrobial classes emerged as independent predictors of invasive infection, although these associations were not consistently supported by statistical analysis [32,33]. Additional associations included recent surgical procedures, ICU admission, mechanical ventilation and immunosuppressive therapy [44]. Case histories indicated that multiple patients had a history of international travel or prior healthcare exposures [34,36–38], reinforcing concerns about cross-border AMR transmission.

## Discussion

In this scoping review, we identified and synthesised fourteen studies reporting CPE within the Caribbean region; an area that, despite its geographic and demographic diversity, remains conspicuously under-represented in global AMR research. The findings illustrate both the increasing detection of CPE and the complexity of resistance mechanisms emerging across the region. Reports from Cuba [31–33], Puerto Rico [40,41], Jamaica [38,39], and Guadeloupe [37] confirm the presence of diverse carbapenemase enzymes, including KPC, NDM-1, NDM-5 and OXA-48, across multiple species of Enterobacterales. The identification of multiple co-producing isolates, such as those harbouring both *bla*_KPC_ and *bla*_NDM_ genes, further suggests local genetic exchange and possible endemic transmission [33].

CPE have become emblematic of the growing threat posed by AMR, a phenomenon that has steadily reduced the effectiveness of some of the most potent antimicrobials available to treat serious infections [3,9,46]. The global spread of CPE has moved beyond a theoretical concern and is now a reality that contributes to increased mortality worldwide. In Brazil, CRE BSIs were associated with a 71.6% fatality rate, compared to 28.4% for susceptible strains [47]. A systematic review and meta-analysis of CRE-BSI that included 14 studies, 13 of which were case reports or series from Latin America (not including countries within the scope of the current study) also reported an overall mortality rate of 34% across 189 paediatric patients [45]. A multicentre European study and US surveillance data both report 30-day mortality rates of up to 44% among patients with invasive CPE infections [48,49]. In China, a multicentre study found a 62% mortality rate for KPC-producing *K. pneumoniae* BSIs [50]. Similarly, infections with NDM-producing strains carry poor prognoses in settings lacking access to newer treatment option [51]. Our review highlights similar concerns in the Caribbean, where mortality associated with CPE infections has been repeatedly documented. In one study from Cuba, for example, two of three patients with KPC-2-producing *K. pneumoniae* died of septic shock [31], while a Jamaican case-study involving NDM-5-producing *K. pneumoniae* resulted in death within 24 hours due to lack of therapeutic options [39]. While in many cases, co-morbidities may have increased risk of poor outcomes, these consistently high mortality rates across diverse settings, including LMICs and SIDS, underscore the need for robust, globally representative surveillance to track CPE burden and guide effective response strategies.

High-risk CPE clones and plasmid-mediated resistance mechanisms, associated with transmission are now documented on nearly every continent [3,52]. Our findings confirm that the Caribbean is no exception. The KPC-producing *K. pneumoniae* ST258 clone, widely disseminated across Europe and the Americas [53], was identified in Curaçao and linked to transfer from Colombia [34]. In the same study, clonal transmission of ST11 KPC-producing *K. pneumoniae* was also demonstrated, mirroring its global role as a high-risk lineage across Asia and Latin America [54]. The global spread of carbapenemases has been further facilitated by highly mobile genetic elements, with IncF, IncA/C and IncX3 plasmids central to the dissemination of NDM enzymes [55,56], and IncL/M plasmids to OXA-48-like enzymes in Europe, North Africa and the Middle East [8]. Caribbean reports similarly reflect these global patterns, with ST11 *K. pneumoniae* in Jamaica carrying NDM-5 on IncX3 and IncFIB plasmids and OXA-48-producing *E. coli* in Guadeloupe linked to IncL/M plasmids [37,38]. These findings highlight the alignment between regional and global dissemination pathways.

The point prevalence study of Thomas et al. 2022 [44], cited an increase in inappropriate broad spectrum antimicrobial use during the SARS-CoV-2 pandemic as contributing to the increased prevalence of CPE with previously undetected carbapenemases and/or co-production of multiple enzymes. This trend has also been reported elsewhere in regions outside the geographic scope of the current study. For example, a study from Argentina in 2024 described the emergence of a CPE epidemiological landscape characterised by equivalent circulation of NDM and KPC, post-COVID [57]. Global trends in the post-COVID period, based on a systematic review, show the widespread emergence of this phenomenon [58]. While the greatest burden is associated with China, India and Iran, for the Caribbean region, this highlights the importance of system-level surveillance and antimicrobial stewardship [59].

The findings of this scoping review reveal that evidence-based literature on the epidemiology of CPE in the region remain sparse, inconsistent, and disproportionately concentrated in a limited number of LMICs and SIDS. Several Caribbean nations, including those with significant population densities and complex healthcare systems such as Haiti, Grenada, Barbados and the Bahamas, have no published reports of CPE. This raises concern that the true burden of resistance may be underestimated [42,43].

The absence of coordinated, region-wide surveillance frameworks for AMR in the Caribbean represents a critical gap in the global response to resistance. Surveillance efforts across the Caribbean are rarely standardised and antimicrobial susceptibility testing (AST) methodologies vary significantly between laboratories. Differences in testing platforms (e.g., disc diffusion, automated systems), interpretive standards (Clinical and Laboratory Standards Institute (CLSI) vs. European Committee on Antimicrobial Susceptibility Testing (EUCAST)) and antimicrobial panels hinder inter-laboratory comparability and undermine regional data integration. Confirmatory testing for carbapenemase production is not routinely performed in many Caribbean settings due to limited access to phenotypic assays (e.g., Carba NP, synergy testing) or molecular diagnostics. Consequently, the detection of CPE is often incidental, triggered by clinical suspicion or unusual resistance patterns, rather than the result of systematic surveillance. This reactive approach delays recognition of transmission events and precludes early containment. Furthermore, the paucity of CPE surveillance in environmental or agricultural settings evidenced here, further highlights a significant knowledge gap that limits the potential for a unified One Health approach to be applied.

Current global initiatives such as GLASS and PAHO Latin American Network for Antimicrobial Resistance Surveillance (ReLAVRA+) network have sought to advance standardised AMR reporting and improve regional surveillance capacity [11,60]. Nonetheless, according to the WHO’s 2025 GLASS report, fewer than half of the countries in the Americas Region submit AMR data regularly, with participation from the Caribbean even more limited [20,61], a disparity that reflects the structural, financial and technical barriers as outlined above. Notably, Trinidad and Tobago, the only Caribbean country contributing data to WHO GLASS and one with relatively established laboratory capacity, was not represented in the included studies. This highlights an important distinction between participation in global surveillance initiatives and the generation of peer-reviewed primary literature, and underscores persistent gaps in the published evidence base despite the presence of surveillance infrastructure. The ReLAVRA+ network has also noted that many Caribbean countries lack national reference laboratories and functional AMR surveillance systems [62]. While some countries (e.g., Cuba) have demonstrated robust local capacity and published surveillance data, many others lack even baseline prevalence figures for critical pathogens.

Underlying these limitations are substantial resource constraints. Many laboratories lack the financial, technical and human resources necessary to implement routine screening for carbapenemase producing organisms [23,63]. Furthermore, the capacity for isolation or cohorting of CPE carriers is limited in many hospitals, making routine screening operationally unfeasible even when laboratory capacity exists. In the absence of adequate IPC infrastructure, such as single rooms and dedicated staff, the ability to act on screening results is severely compromised [23,64]. Additionally, AMS programmes, where they exist, are often under-resourced or restricted to tertiary-care hospitals, with little oversight in community settings [65,66]. Moreover, the overuse and inappropriate prescribing of antimicrobials in both hospital and outpatient settings is driven by weak regulatory frameworks, widespread non-prescription access to antimicrobials and limited clinician access to diagnostic microbiology [67–69]. These interlinked deficiencies create an environment in which CPE can emerge, persist and spread largely unchecked, highlighting the urgent need for system-wide investment in diagnostic, AMS, regulatory and IPC capacity.

The Caribbean does not represent a single, coherent regulatory or AMS environment, and there is considerable variation between territories that is likely to shape AMR dynamics [70]. Some jurisdictions, including French overseas departments such as Guadeloupe and Martinique, and certain UK overseas territories, operate within European regulatory systems where antimicrobial prescribing is more tightly governed and non-prescription access is restricted [71,72]. In contrast, across a number of independent Caribbean nations, regulatory enforcement is more variable, and over-the-counter access to antimicrobials and informal distribution channels remain documented challenges [67,73]. These differences are likely to influence antimicrobial exposure at population level, and therefore the selective pressures that drive the emergence and persistence of resistant organisms, including CPE [74]. It follows that some of the variation observed in carbapenemase types, clonal lineages, and reported burden across the region may reflect these underlying structural differences, rather than purely epidemiological factors.

The global response to AMR coalesced with the adoption of the WHO Global Action Plan (GAP) on AMR, endorsed by all 194 Member States. The plan outlined five strategic objectives, including improved surveillance, optimised antimicrobial use and investment in innovation. Crucially, it also formalised the One Health approach, recognising the interconnectedness of human, animal, and environmental health in driving AMR. This cross-sectoral model is especially relevant for regions like the Caribbean, where health, agriculture and environmental systems are closely intertwined.

While over 170 countries globally have developed National Action Plans (NAPs) on AMR, progress within the Caribbean has been uneven. As of 2024, ten of the Caribbean Public Health Agency (CARPHA) 26 member states have formalised AMR action plans, and only a limited number, including Barbados, Trinidad and Tobago, and the Cayman Islands, have explicitly incorporated a One Health framework. Several others, such as Jamaica, Guyana and Saint Lucia, are in various stages of development or early implementation, often with technical support from PAHO, CARPHA and the Fleming Fund [75,76]. However, full enactment remains limited, with persistent challenges in cross-sectoral coordination.

In an era of intensified globalisation, high-volume travel and increasingly interconnected healthcare systems, effective AMR containment efforts must be globally representative. The ability of any single country to prevent or control the spread of AMR pathogens is inherently tied to the capacities of others, particularly in regions characterised by high cross-border mobility like the Caribbean. Recognising this interdependence, recent global frameworks have called not only for reductions in AMR-associated mortality, but also for the expansion of equitable access to diagnostics, laboratory strengthening and surveillance integration across LMICs and SIDS. These goals reflect a shift in emphasis from isolated national responses to collective international responsibility, acknowledging that pathogens such as CPE thrive on the absence of coordinated action. In this context, under-resourced regions must not remain peripheral to the global AMR agenda; rather, they require targeted support to develop functional AMR surveillance systems, strengthen IPC infrastructure, and generate timely, actionable AMR data. Failure to do so risks persistence of global blind spots, allowing AMR to emerge unchecked and silently disseminate through international networks of travel and healthcare.

## Conclusion

This scoping review synthesises the limited published data on CPE across the Caribbean, confirming the presence of major carbapenemases, including KPC, NDM and OXA-48, in multiple countries and high-risk clones. However, the overall volume of published research remains limited and unevenly distributed, highlighting critical gaps in AMR surveillance and reporting.

The available evidence indicates that CPE detection has been reported almost exclusively from human healthcare settings, largely in the context of clinical infection and, less frequently, colonisation. Only a single included study identified CPE in an environmental setting, and no eligible studies reported animal reservoirs. This pattern is more likely to reflect limitations in surveillance coverage rather than true absence of non-human reservoirs, and underscores the need for a more integrated One Health approach to AMR surveillance in the Caribbean.

Strengthening laboratory capacity, standardising detection practices and fostering regional and international collaboration are essential to ensure timely identification, containment, and mitigation of CPE. Given that AMR continues to transcend national borders, an effective globally informed response must include data from historically under-represented regions including the Caribbean.

## Supporting information

S1 Appendix Included countries and full electronic search strategies for MEDLINE (PubMed), EMBASE, Cochrane CENTRAL and Web of Science, including all search terms, combinations and numbers of records retrieved. (DOCX)

S1 Checklist Completed Preferred Reporting Items for Systematic Reviews and Meta-Analyses extension for Scoping Reviews (PRISMA-ScR) checklist [28,30], indicating the manuscript pages on which each reporting item is addressed. (PDF)
