# Supplementary material for: The Caribbean under the radar? A scoping review of carbapenemase-producing Enterobacterales across the region
Source: PLOS Glob Public Health. 2026 Aug 26;6(8):e0007176. doi: 10.1371/journal.pgph.0007176 (PMC13513946; doi:10.1371/journal.pgph.0007176)
Supplement: Supplementary file 2 [file pgph.0007176.s001.docx]

**Appendix**

List of included countries:

1. Anguilla
2. Antigua and Barbuda
3. Aruba
4. Bahamas
5. Barbados
6. Belize
7. Bonaire
8. Cuba
9. Curaçao
10. Dominica
11. Dominican Republic
12. Grenada
13. Guadeloupe and Martinique
14. Haiti
15. Jamaica
16. Puerto Rico
17. Saint Kitts and Nevis
18. Saint Lucia
19. Saint Vincent and the Grenadines
20. Trinidad and Tobago

Search strategies:

MEDLINE (via PubMed)

| Search Number | Search Query | Results |
| --- | --- | --- |
| #4 | #1 AND #2 AND #3 | 137 |
| #3 | "Caribbean Region"[Mesh OR caribbean OR "jamaica" OR "puerto rico" OR "cuba" OR "guadeloupe" OR "martinique" OR "dominican republic" OR "trinidad" OR "tobago" OR "barbados" OR "antigua" OR "barbuda" OR "st lucia" OR "dominica" OR "grenada" OR "st vincent" OR "grenadines" OR "haiti" OR "bahamas" OR "st kitts" OR "nevis" OR "belize" OR "anguilla" OR "curacao" OR "bonaire" OR "aruba" OR "caricom" | 156,389 |
| #2 | "beta-Lactamases"[Mesh] OR carbapenemase* OR "carbapenemase-producing" OR "carbapenemase-producing enterobacteriaceae" OR "carbapenemase-producing enterobacterales" OR KPC OR NDM OR OXA-48 OR VIM OR IMP | 58,150 |
| #1 | "Enterobacteriaceae"[Mesh] OR enterobacterales OR enterobacteriaceae OR klebsiella OR "escherichia coli" OR "e. coli" OR enterobacter OR serratia OR citrobacter OR morganella | 638,570 |

EMBASE

| Search Number | Search Query | Results |
| --- | --- | --- |
| #4 | #1 AND #2 AND #3 | 136 |
| #3 | 'caribbean'/exp OR caribbean OR 'jamaica' OR 'puerto rico' OR 'cuba' OR 'guadeloupe' OR 'martinique' OR 'dominican republic' OR 'trinidad' OR 'tobago' OR 'barbados' OR 'antigua' OR 'barbuda' OR 'st lucia' OR 'dominica' OR 'grenada' OR 'st vincent' OR 'grenadines' OR 'haiti' OR 'bahamas' OR 'st kitts' OR 'nevis' OR 'belize' OR 'anguilla' OR 'curacao' OR 'bonaire' OR 'aruba' OR 'caricom' | 256,329 |
| #2 | 'carbapenemase'/exp OR carbapenemase* OR cpe OR kpc OR ndm OR 'oxa 48' OR vim OR imp | 132,179 |
| #1 | 'enterobacterales'/exp OR enterobacterales OR enterobacteriaceae OR klebsiella OR escherichia OR 'e. coli' OR enterobacter OR serratia OR citrobacter OR morganella | 835,926 |

Cochrane CENTRAL

| Search Query | Results |
| --- | --- |
| ("enterobacterales" OR "enterobacteriaceae" OR "klebsiella" OR "escherichia coli" OR "e. coli" OR "enterobacter" OR "serratia" OR "citrobacter" OR "morganella") AND  ("caribbean OR "jamaica" OR "puerto rico" OR "cuba" OR "guadeloupe" OR "martinique" OR "dominican republic" OR "trinidad" OR "tobago" OR "barbados" OR "antigua" OR "barbuda" OR "st lucia" OR "dominica" OR "grenada" OR "st vincent" OR "grenadines" OR "haiti" OR "bahamas" OR "st kitts" OR "nevis" OR "belize" OR "anguilla" OR "curacao" OR "bonaire" OR "aruba" OR "caricom") | 0 |

Web of Science

| Search Number | Search Query | Results |
| --- | --- | --- |
| #4 | #1 AND #2 AND #3 | 89 |
| #3 | "caribbean" OR "jamaica" OR "puerto rico" OR "cuba" OR "guadeloupe" OR "dominican republic" OR "trinidad" OR "barbados" OR "antigua" OR "st lucia" OR "grenada" OR "st vincent" OR "haiti" OR "bahamas" OR "st kitts" OR "belize" OR "anguilla" OR "curacao" OR "aruba" OR "caricom" | 379,761 |
| #2 | "carbapenemase" OR "carbapenemase-producing" OR CPE OR KPC OR NDM OR OXA-48 OR VIM OR IMP | 107,212 |
| #1 | "enterobacterales" OR "enterobacteriaceae" OR "klebsiella" OR "escherichia coli" OR "e. coli" OR "enterobacter" OR "serratia" OR "citrobacter" OR "morganella" | 717,105 |
