# Supplementary material for: The Caribbean under the radar? A scoping review of carbapenemase-producing Enterobacterales across the region
Source: PLOS Glob Public Health. 2026 Aug 26;6(8):e0007176. doi: 10.1371/journal.pgph.0007176 (PMC13513946; doi:10.1371/journal.pgph.0007176)
Supplement: Supplementary file 3 [file pgph.0007176.s004.docx]

Department of Clinical Microbiology,

Royal College of Surgeons in Ireland,

Dublin, Republic of Ireland.

23^rd^ January 2026

Dear Editor,

Thank you for the opportunity to revise our manuscript entitled, “The Caribbean under the radar? A scoping review of carbapenemase-producing Enterobacterales across the region (Manuscript ID: PGPH-D-25-03527).” We are grateful to you and the reviewers for the time and thoughtful feedback provided.

We have carefully considered all editorial and reviewer comments and have revised the manuscript accordingly. A detailed, point-by-point response to each comment is provided below. For ease of review, all responses to comments are highlighted in blue, and corresponding changes have been incorporated into the revised manuscript.

In summary, we have:

- Revised the Financial Disclosure statement to ensure full compliance with journal requirements;
- Updated Table 1 to improve clarity, including refinement of key observations, inclusion of denominators where available, explicit distinction between phenotypic and molecular diagnostic methods, and clearer reporting of isolate sources;
- Expanded the conclusion to better contextualise findings across clinical and environmental reservoirs within a One Health framework, while remaining faithful to the available evidence;
- Uploaded figures and supporting information in accordance with journal formatting requirements and clarified data availability.

We believe these revisions have strengthened the clarity, methodological transparency, and public health relevance of the manuscript, and we hope it is now suitable for further consideration.

Thank you again for your consideration.

Kind regards,

Saied Ali
On behalf of the authors

**Response to Reviewers – Manuscript PGPH-D-25-03527**

**Editorial Comments**

1. Please amend your detailed Financial Disclosure statement. This is published with the article. It must therefore be completed in full sentences and contain the exact wording you wish to be published.
i. Please clarify all sources of financial support for your study. List the grants, grant numbers, and organizations that funded your study, including funding received from your institution. Please note that suppliers of material support, including research materials, should be recognized in the Acknowledgements section rather than in the Financial Disclosure.
ii. State the initials, alongside each funding source, of each author to receive each grant. For example: "This work was supported by the National Institutes of Health (####### to AM; ###### to CJ) and the National Science Foundation (###### to AM)."
iii. State what role the funders took in the study. If the funders had no role in your study, please state: “The funders had no role in study design, data collection and analysis, decision to publish, or preparation of the manuscript.”
iv. If any authors received a salary from any of your funders, please state which authors and which funders.

We thank the reviewer for highlighting the need to amend the Financial Disclosure statement. We have revised this section to ensure it is written in full sentences, uses publication-ready wording, and fully complies with the journal’s requirements.

The revised Financial Disclosure now explicitly identifies the funding source, grant number and awarding organisation, specifies the author who received the award, and clearly states the role of the funders. Specifically, the manuscript now states that the study was funded by the Health Research Board (ICAT-2022-001) through the ICAT Programme, with the award held by SA. The supporting organisations of the ICAT Programme are also listed in full.

In line with the journal’s guidance, we additionally confirm that the funders had no role in study design, data collection and analysis, decision to publish, or preparation of the manuscript, and that no salaries or honoraria were provided to any named authors by the funders.

No material suppliers or commercial entities contributed to this work, and therefore no additional acknowledgements were required.

2. Please upload separate figure files in .tif or .eps format. Also, remove the figures from your manuscript file but keep the legends.

For more information about figure files please see our guidelines:

<https://journals.plos.org/globalpublichealth/s/figures> 
[https://journals.plos.org/globalpublichealth/s/figures#loc-file-requirements](https://eur02.safelinks.protection.outlook.com/?url=https%3A%2F%2Fjournals.plos.org%2Fglobalpublichealth%2Fs%2Ffigures%23loc-file-requirements&data=05%7C02%7Csaiedali%40rcsi.ie%7C16e4d66e7fbe4dac21ba08de4ea0969d%7C607041e7a8124670bd3030f9db210f06%7C0%7C0%7C639034648799008101%7CUnknown%7CTWFpbGZsb3d8eyJFbXB0eU1hcGkiOnRydWUsIlYiOiIwLjAuMDAwMCIsIlAiOiJXaW4zMiIsIkFOIjoiTWFpbCIsIldUIjoyfQ%3D%3D%7C0%7C%7C%7C&sdata=RqiiBa6z0rBfem7HHeOwL5l56gvG0TFJOY7O756JSnM%3D&reserved=0)

In accordance with the journal’s submission requirements, the figure has now been uploaded as a separate file in the required format. Figure 1 (PRISMA flow diagram of search strategy and study selection) has been provided as a standalone .tif file. The figure has been removed from the main manuscript file, while the corresponding figure legend has been retained.

3. We have noticed that you have uploaded Supporting Information files, but you have not included a list of legends. Please add a full list of legends for your Supporting Information files after the references list.

In accordance with the journal’s requirements, a full list of legends for the Supporting Information has now been added after the references section. The manuscript now includes the following entry:

*S1 File. Full electronic search strategies for MEDLINE (PubMed), EMBASE, Cochrane CENTRAL and Web of Science, including all search terms, combinations and numbers of records retrieved.*

4. We note that your Data Availability Statement is currently as follows: All data underlying the results are fully available in the manuscript and no additional datasets were generated or required. Please confirm at this time whether or not your submission contains all raw data required to replicate the results of your study. Authors must share the “minimal data set” for their submission. PLOS defines the minimal data set to consist of the data required to replicate all study findings reported in the article, as well as related metadata and methods ([https://journals.plos.org/plosone/s/data-availability#loc-minimal-data-set-definition](https://eur02.safelinks.protection.outlook.com/?url=https%3A%2F%2Fjournals.plos.org%2Fplosone%2Fs%2Fdata-availability%23loc-minimal-data-set-definition&data=05%7C02%7Csaiedali%40rcsi.ie%7C16e4d66e7fbe4dac21ba08de4ea0969d%7C607041e7a8124670bd3030f9db210f06%7C0%7C0%7C639034648799023379%7CUnknown%7CTWFpbGZsb3d8eyJFbXB0eU1hcGkiOnRydWUsIlYiOiIwLjAuMDAwMCIsIlAiOiJXaW4zMiIsIkFOIjoiTWFpbCIsIldUIjoyfQ%3D%3D%7C0%7C%7C%7C&sdata=lpfZGc%2FaQHh60rgSmHqArVeen90qnj4Ko%2B3IPes2Jdg%3D&reserved=0)).
    
    For example, authors should submit the following data:
    - The values behind the means, standard deviations and other measures reported;
    - The values used to build graphs;
    - The points extracted from images for analysis.
    
    Authors do not need to submit their entire data set if only a portion of the data was used in the reported study. If your submission does not contain these data, please either upload them as Supporting Information files or deposit them to a stable, public repository and provide us with the relevant URLs, DOIs, or accession numbers. For a list of recommended repositories, please see [https://journals.plos.org/plosone/s/recommended-repositories](https://eur02.safelinks.protection.outlook.com/?url=https%3A%2F%2Fjournals.plos.org%2Fplosone%2Fs%2Frecommended-repositories&data=05%7C02%7Csaiedali%40rcsi.ie%7C16e4d66e7fbe4dac21ba08de4ea0969d%7C607041e7a8124670bd3030f9db210f06%7C0%7C0%7C639034648799038222%7CUnknown%7CTWFpbGZsb3d8eyJFbXB0eU1hcGkiOnRydWUsIlYiOiIwLjAuMDAwMCIsIlAiOiJXaW4zMiIsIkFOIjoiTWFpbCIsIldUIjoyfQ%3D%3D%7C0%7C%7C%7C&sdata=lVRSDoE73tDlPnR7NRi0tTXOuX5BG0dzZLxOlTq7vgs%3D&reserved=0).

If there are ethical or legal restrictions on sharing a de-identified data set, please explain them in detail (e.g., data contain potentially sensitive information, data are owned by a third-party organization, etc.) and who has imposed them (e.g., an ethics committee). Please also provide contact information for a data access committee, ethics committee, or other institutional body to which data requests may be sent. If data are owned by a third party, please indicate how others may request data access.

If the reviewer comments include a recommendation to cite specific previously published works, please review and evaluate these publications to determine whether they are relevant and should be cited. There is no requirement to cite these works unless the editor has indicated otherwise.

We confirm that the submission contains all data required to replicate the results of this study. As this is a scoping review, all data underlying the findings were extracted from published studies and are fully reported within the manuscript (including Table 1, Figure 1, and the Results section). No primary or unpublished datasets were generated as part of this work.

The complete electronic search strategy used to identify eligible studies, including all databases searched, search terms, combinations and numbers of records retrieved, is provided in S1 File, enabling full replication of the study identification and selection process. No additional numerical datasets, graph source data, or image-derived data were generated beyond those presented in the manuscript and Supporting Information.

We further note that all reviewers have confirmed that the data underlying the findings are fully available, in line with the PLOS Data Policy (Reviewer #1: Yes; Reviewer #2: Yes; Reviewer #3: Yes).

There are no ethical or legal restrictions on data sharing for this study, as all data are derived from published literature.

**Reviewer Comments**

Reviewer #1

This is a relevant and timely topic, and the authors successfully compile and summarize current information on CPE in the Caribbean. The manuscript is generally well written, with a clear structure and a methodology appropriate for a review article. The abstract and introduction provide sufficient context and highlight the importance of the subject within public health.

We thank the reviewer for these positive and encouraging comments. We are grateful for their assessment that the topic is relevant and timely, and that the manuscript provides a clear and well-structured synthesis of the available evidence on carbapenemase-producing Enterobacterales in the Caribbean. We also appreciate their recognition that the methodology is appropriate for a review article and that the abstract and introduction provide sufficient public health context. These comments are very helpful and affirm the overall framing and approach of the manuscript.

However, Table 1 appears excessively detailed and could benefit from a more concise presentation. In particular, the “Key Observations” column contains extensive descriptive text that could be reduced to the essential points.

We thank the reviewer for this helpful suggestion. We agree that the “Key Observations” column in Table 1 contained more narrative detail than was necessary for a summary table. To improve clarity and readability, we have now edited this column to retain only the essential findings from each study, focusing on epidemiological relevance, key molecular features and clinically important outcomes.

In the “Number of CPE Isolates” column, it would also be helpful to include, when available, the total number of isolates tested in each study, as this would improve comparability across the included works.

Where reported in the primary studies, we have revised the table to include the total number of isolates screened alongside the number of confirmed CPE isolates. To reflect this, the column heading has been amended to “Number of CPE Isolates / Total Number of Isolates Tested.” For large surveillance studies, denominators are now explicitly reported.

However, many included studies, particularly case reports, outbreak investigations, environmental surveys and narrative reviews, did not report a defined total number of isolates screened. In these instances, denominators could not be inferred without introducing misleading assumptions. This limitation reflects the heterogeneity of the underlying literature and is inherent to scoping reviews of under-surveilled regions.

Additionally, when the table lists “various” under “Detection & Diagnostic Methods,” it would be important to specify whether these methods are molecular or phenotypic. This distinction directly affects detection sensitivity and reliability, since a carbapenem-resistant phenotype does not necessarily indicate carbapenemase production, and conversely, some carbapenemase producers (e.g., OXA-48-like enzymes) may appear susceptible in vitro while still being clinically relevant.

We thank the reviewer for this important methodological point. We agree that distinguishing between phenotypic and molecular detection methods is essential, given the variable sensitivity of phenotypic assays and the potential for carbapenemase producers, particularly OXA-48–like enzymes, to appear carbapenem-susceptible in vitro.

To address this, we have revised Table 1 to remove the term “various” and replaced it with explicit descriptions indicating whether detection relied on phenotypic susceptibility testing, phenotypic carbapenemase confirmation assays, molecular methods (PCR and/or sequencing), or combinations thereof.

Another aspect that requires clarification is the type of samples included in each study—whether environmental, human (clinical or colonization), or animal. Although the text briefly mentions that one study is environmental and others clinical, this information is not consistently reflected in Table 1 nor fully discussed in the manuscript. Explicitly identifying the source of the isolates is essential for understanding transmission dynamics and epidemiological significance.

We agree that isolate source is critical for understanding transmission dynamics. Rather than introducing an additional column, which would risk oversimplification given the mixed and inconsistently reported sampling frameworks across studies, we have incorporated source information directly into the “Number of CPE Isolates / Total Number of Isolates Tested” columns.

Where primary studies explicitly reported numerical differentiation between clinical infection and colonisation isolates, this information has been incorporated into Table 1. However, many studies did not provide a clear numerical breakdown by isolate source. In such cases, we have transparently indicated mixed clinical and colonisation populations rather than inferring counts not reported in the original publications.

Because this information is missing, the conclusion also does not address how carbapenemase-producing Enterobacterales are distributed across different reservoirs. Expanding the conclusion to discuss the implications of detecting these resistances in clinical, environmental, and potentially animal samples would strengthen the manuscript, particularly from a One Health perspective, given the interconnectedness of human, animal, and environmental health in the spread of antimicrobial resistance.

We thank the reviewer for raising this point. We have revised the Conclusion to explicitly describe the distribution of CPE across reported reservoirs (Lines 412-421). The evidence identified in this review shows that CPE detection in the Caribbean has been reported almost exclusively from human clinical settings, with limited data from environmental sources and no eligible studies describing animal reservoirs. We now highlight this imbalance as a limitation of the current evidence base and as an important gap for future One Health–aligned surveillance and research (Lines 418-421).

Overall, the manuscript has strong potential, and addressing the points above would further enhance its clarity.

We thank the reviewer for this positive and constructive assessment. We have addressed the points raised above and believe that the revisions have improved the clarity and presentation of the manuscript.

**Reviewer #2**

The manuscript is reviewing in a standardised way the research published on CPE in the Caribbean area. From the work published it appears as a geographic area that is not sufficiently in focus to assess properly the level of antimicrobial resistance in Enterobacterales. Hopefully this manuscript will draw attention to researchers in the area to publish more information on AMR in this geographic region.

We agree with the reviewer’s observation. By applying a standardised approach to synthesising the published literature on carbapenemase-producing Enterobacterales in the Caribbean, this manuscript highlights the relatively limited volume of available data from this region. This may constrain the ability to fully characterise the epidemiology and burden of antimicrobial resistance in Enterobacterales. We hope that this review may help to draw attention to the need for further reporting and research in this geographic area.

**Reviewer #3**

The authors reported a literature review on the occurrence of carbapenemases in Caribbean area. The study offers an interesting prospective of the carbapenem resistance burden but the results are not properly narrated. The structure of narration just reported a list of the literature findings, without a clear discussion on the impact of such findings. Moreover, to better understand the impact of such circulation, information regarding plasmids types should be included. I suggest a re-evaluation of the type of narration and presentation of literature findings.

We thank the reviewer for this comment and for recognising the relevance of the topic. We respectfully disagree that the Results section is presented merely as a list of individual study findings. In accordance with PRISMA-ScR guidance, the Results are structured as a thematic narrative synthesis, integrating evidence across studies by geographic distribution, bacterial species, carbapenemase type, molecular epidemiology, antimicrobial susceptibility profiles and source of isolates.

The interpretation and broader public health implications of these findings are intentionally addressed in the Discussion section, where the results are contextualised within the global epidemiology of carbapenemase-producing Enterobacterales, including comparisons with international mortality data, transmission dynamics, surveillance gaps and implications for infection prevention and antimicrobial stewardship.

With regard to plasmid data, information on plasmid types is already included where reported in the primary literature. Plasmid-mediated resistance is described in both the Results and Discussion sections, including identification of IncX3, IncFIB, IncR, IncA/C, IncHI1B and IncL/M plasmids, and their association with high-risk international clones such as ST11 and ST258 (Lines 239-241, 321-327). However, plasmid-level characterisation was not performed or reported in many Caribbean studies, and this limitation is explicitly acknowledged and discussed as a key gap in the existing evidence base.
