# Supplementary material for: The Caribbean under the radar? A scoping review of carbapenemase-producing Enterobacterales across the region
Source: PLOS Glob Public Health. 2026 Aug 26;6(8):e0007176. doi: 10.1371/journal.pgph.0007176 (PMC13513946; doi:10.1371/journal.pgph.0007176)
Supplement: Supplementary file 4 [file pgph.0007176.s005.docx]

Department of Clinical Microbiology,

Royal College of Surgeons in Ireland,

Dublin, Republic of Ireland.

17^th^ March 2026

Dear Editor,

Thank you for the opportunity to revise our manuscript entitled, “The Caribbean under the radar? A scoping review of carbapenemase-producing Enterobacterales across the region” (Manuscript ID: PGPH-D-25-03527). We are grateful to you and the reviewers for the time and thoughtful feedback provided.

We have carefully considered all reviewer comments and have revised the manuscript accordingly. A detailed, point-by-point response to each comment is provided below. For ease of review, all responses are presented in blue, with corresponding changes tracked in the revised manuscript and line numbers provided at each reviewer response.

In summary, we have:

- Clarified the geophysical definition of the Caribbean and explicitly referenced the included countries and territories in the Supplementary Material to improve transparency and reproducibility;
- Expanded the Discussion to better reflect heterogeneity in antimicrobial stewardship and regulatory frameworks across the Caribbean and its implications for AMR dynamics;
- Standardised carbapenemase nomenclature throughout the manuscript and corrected the classification of OXA-72;
- Incorporated additional contextual interpretation regarding the relationship between surveillance participation (e.g. WHO GLASS) and the availability of peer-reviewed evidence;
- Corrected the typographical error in the PRISMA flow diagram

We believe these revisions have strengthened the clarity, scientific accuracy, and contextual depth of the manuscript, and we hope it is now suitable for further consideration.

Thank you again for your consideration.

Kind regards,

Saied Ali

On behalf of the authors

**Response to Reviewers – Manuscript PGPH-D-25-03527 (Second Round)**

**Reviewer #1**

The article is well written and well organized. The requested corrections have been properly addressed, and the authors have carefully considered and responded to the questions raised during the review process. The revisions have improved the clarity and overall quality of the manuscript. In its current form, the article is suitable for publication.

We thank the reviewer for their positive and encouraging assessment of our manuscript. We appreciate their recognition of the revisions undertaken and are pleased that the changes have improved the clarity and overall quality of the work.

**Reviewer #3**

The comments were not fully followed and the narrative was not improved as requested.

We thank the reviewer for their reassessment of the manuscript. However, we respectfully disagree with the characterisation of the Results as a simple list of study findings or that the narrative has not been improved. The Results section has been deliberately structured as a thematic narrative synthesis in line with PRISMA-ScR guidance, integrating findings across studies by key domains including geographic distribution, species, carbapenemase types, molecular epidemiology, and sources of isolates. This approach is consistent with established scoping review methodology and avoids over-interpretation within the Results section.

The separation between evidence presentation and interpretation has been applied intentionally. As recommended in reporting frameworks, the Results focus on mapping the available evidence, while the Discussion provides critical interpretation of the findings, including their implications for transmission dynamics, surveillance gaps, and infection prevention and control. This structure has also been considered appropriate by other reviewers.

In light of this, we consider that the current structure and narrative are methodologically appropriate and aligned with reporting standards.

**Reviewer #4**

1. Definition of Caribbean and Its Implications

The geophysical definition is defensible but creates some anomalies worth acknowledging. Under this definition, Trinidad and Tobago and some mainland coastal territories may be included or excluded in ways that are not entirely transparent to readers unfamiliar with the region's geography. A brief supplementary note clarifying exactly which 20 nations/territories were included and which borderline cases were considered would improve.

We thank the reviewer for this comment. While we maintain that a geophysical definition based on the Caribbean tectonic plate is methodologically appropriate for this review, we recognise that this may not be immediately intuitive to all readers. To address this, we have clarified the definition in the manuscript and ensured that the full list of the 20 included countries and territories is explicitly provided in the Supplementary Material. Inclusion of borderline mainland territories was determined based on tectonic plate boundaries, and this has now been more clearly articulated to improve transparency and reproducibility.

See Lines 163-168 – “This definition encompasses 20 countries and territories, including island states and selected mainland regions located on the Caribbean plate. As such, inclusion may differ from conventional geopolitical definitions of the Caribbean, particularly for mainland territories that are geographically part of continental landmasses but lie on the Caribbean tectonic plate. The full list of included countries and territories is provided in the Search Strategy section of the Appendix.”

Trinidad and Tobago — the only Caribbean country submitting data to WHO GLASS, and one with a relatively functional reference laboratory system — is entirely absent from the included studies despite being a logical candidate for CPE detection. The authors should acknowledge this paradox directly: GLASS participation does not guarantee published primary literature.

We thank the reviewer for this important observation. We agree that the absence of Trinidad and Tobago from the included studies, despite being the only Caribbean country contributing data to WHO GLASS and having relatively established laboratory capacity, represents a notable and informative finding. In response, we have now explicitly acknowledged this in the Discussion. We highlight that participation in global surveillance initiatives such as GLASS does not necessarily translate into the generation of peer-reviewed primary literature. This distinction underscores an important gap between surveillance activity and published evidence, and supports our broader finding that data on carbapenemase-producing Enterobacterales in the Caribbean remain limited and unevenly distributed despite the presence of surveillance infrastructure in some settings.

See Lines 358-362 – “Notably, Trinidad and Tobago, the only Caribbean country contributing data to WHO GLASS and one with relatively established laboratory capacity, was not represented in the included studies. This highlights an important distinction between participation in global surveillance initiatives and the generation of peer-reviewed primary literature, and underscores persistent gaps in the published evidence base despite the presence of surveillance infrastructure.”

2.Inconsistency in Carbapenemase Nomenclature and Enzyme Classification
Throughout the manuscript, OXA-48 is referred to inconsistently — sometimes as "OXA-48" and elsewhere implicitly as "OXA-48-like." This distinction matters scientifically, as OXA-48-like enzymes include OXA-181, OXA-232, and others with differing hydrolytic profiles and epidemiological origins. The Dominican Republic environmental study detected OXA-72, which is an OXA-24/40-like enzyme (not OXA-48 producers as described at line 230-231:"OXA-48 producers were also reported in multi-country Caribbean studies and in environmental samples from the Dominican Republic").

We thank the reviewer for this important observation regarding the distinction between OXA-48 and OXA-48-like carbapenemases. We agree that this distinction is scientifically relevant, as OXA-48-like enzymes (e.g. OXA-181, OXA-232) differ in their hydrolytic activity and epidemiological distribution. In response, we have reviewed the manuscript throughout and standardised the nomenclature to ensure consistent and accurate use of “OXA-48-like” where appropriate, while retaining “OXA-48” only where specifically reported in the primary studies. We also acknowledge and apologise for the misclassification of OXA-72 in the original text. On initial review, OXA-72 was described as an OXA-48 enzyme in a published study (doi: 10.3390/antibiotics10010089), which informed our wording. Following the reviewer’s comment, we re-examined this classification and have corrected it accordingly. OXA-72 is now appropriately described as an OXA-24/40-like carbapenemase, and the relevant section of the manuscript has been amended to reflect this distinction.

See Lines 234-236 – “OXA-48 and OXA-48-like producers were also reported in multi-country Caribbean studies, while environmental samples from the Dominican Republic identified OXA-72, an OXA-24/40-like carbapenemase.[34,41–44]”

3. Antimicrobial Stewardship and Regulatory Context

The discussion correctly identifies weak AMS frameworks and non-prescription antibiotic access as drivers of resistance, but the treatment is superficial. The Caribbean presents a highly fragmented regulatory landscape: some territories (UK Overseas Territories, French Departments like Guadeloupe and Martinique) operate under metropolitan European regulatory frameworks with prescription requirements, while independent nations vary enormously. This regulatory heterogeneity has direct consequences for selection pressure and CPE emergence, yet the authors treat the region as broadly homogeneous in this regard. Guadeloupe's regulatory environment, for instance, is materially different from Jamaica's or Haiti's, and this likely explains some of the epidemiological differences observed.

We thank the reviewer for this important and insightful comment. We agree that the Caribbean is not a homogeneous regulatory environment and that substantial heterogeneity exists across territories. In response, we have revised the Discussion to better reflect this complexity. We now explicitly acknowledge the variation in antimicrobial regulatory frameworks across the region, including differences in prescription requirements, enforcement, and access to antimicrobials. We further emphasise that this heterogeneity is likely to influence antimicrobial selection pressure and may contribute to observed differences in the epidemiology of carbapenemase-producing Enterobacterales across the region, as highlighted.

See Lines 383-395 – “The Caribbean does not represent a single, coherent regulatory or AMS environment, and there is considerable variation between territories that is likely to shape AMR dynamics.[66] Some jurisdictions, including French overseas departments such as Guadeloupe and Martinique, and certain UK overseas territories, operate within European regulatory systems where antimicrobial prescribing is more tightly governed and non-prescription access is restricted.[67,68] In contrast, across a number of independent Caribbean nations, regulatory enforcement is more variable, and over-the-counter access to antimicrobials and informal distribution channels remain documented challenges.[63,69] These differences are likely to influence antimicrobial exposure at population level, and therefore the selective pressures that drive the emergence and persistence of resistant organisms, including CPE.[70] It follows that some of the variation observed in carbapenemase types, clonal lineages, and reported burden across the region may reflect these underlying structural differences, rather than purely epidemiological factors.”

4. The PRISMA Flow Diagram Contains an Arithmetical Inconsistency
The flow diagram states 362 records identified but lists 137 (MEDLINE) + 636 (EMBASE) + 89 (Web of Science) = 862 records — not 362. This is a significant inconsistency that need to clarify. Does 362 records were screened from 862 records or otherwise?

We thank the reviewer for highlighting this inconsistency. This was a typographical error in the figure, where the number of records identified through EMBASE was incorrectly reported as 636 instead of 136. The correct total number of records identified is therefore 362, as originally stated. This discrepancy was limited to the flow diagram and did not affect the screening or analysis. The correct figures were consistently reported in the Supplementary Material (Appendix). The flow diagram has now been amended to reflect the accurate values.
