# Supplementary material for: The Caribbean under the radar? A scoping review of carbapenemase-producing Enterobacterales across the region
Source: PLOS Glob Public Health. 2026 Aug 26;6(8):e0007176. doi: 10.1371/journal.pgph.0007176 (PMC13513946; doi:10.1371/journal.pgph.0007176)
Supplement: Supplementary file 5 [file pgph.0007176.s006.docx]

**Response to Editor’s Comments – 3^rd^ Revision**

13^th^ May 2026

Dear Dr Bashar Haruna Gulumbe,

Thank you for the opportunity to further address your editorial comments relating to our revised manuscript. We appreciate your careful review of the submission and the additional guidance provided following the previous revision. We have carefully considered each of the points raised and have revised the manuscript further to improve methodological clarity, internal consistency, and overall presentation.

Please find our point-by-point responses below in blue.

- The authors have made a clear effort to revise the manuscript, and the paper addresses an important and underrepresented public health issue with potential value. However, several issues still need to be resolved before it can be considered further.

We are pleased that our efforts in revising the manuscript are acknowledged and overall, it is viewed positively. We are happy to address the remaining salient issues below.

1. The review methods and the included evidence are not yet fully aligned. The Methods state that only primary studies were eligible, but the included studies and Table 1 also contain narrative reviews and a systematic review/meta-analysis. This needs to be clarified and handled consistently THROUGHOUT the manuscript. I recommend that the authors review this table closely.

Thank-you for these observations of some additional discrepancies. We have now made changes to the text and the table so the evidence is aligned to the methodologies used. As the Scoping Review methodology facilitates inclusion of secondary or supplemental data where appropriate and justifiable, we have corrected this omission from the methods and explained our reasoning. Where this methodology has returned such data (e.g., two narrative reviews) we have explicitly stated that they represent supplemental data and not primary. In light of this further scrutiny, we excluded one publication, a systematic review, and have explained the exclusion in the flowchart. In this case, exclusion was because the primary sources reported, did not originate from the Caribbean nations specified in our methodology.

See tracked changes in the revised manuscript and Table 1

*‘In addition, to align with the objective of mapping the available evidence where context-specific gaps existed (i.e., fragmented research, under-resourcing in the region), systematic reviews, narrative reviews and case reports/series were included in the search. These were included as sources of secondary data (systematic reviews) or supplemental evidence to provide contextual insights (narrative reviews) or to highlight the trajectory of emerging CPE strains in the region (case reports) [Lines 175-180].’*

2. The study selection and PRISMA reporting also require further correction. Although the earlier EMBASE typo was explained, the flow diagram still does not read cleanly, particularly in how duplicates, screened records, full-text exclusions, and included studies are accounted for across stages. The numbers and exclusion categories should be presented in a way that is fully transparent and internally consistent.

We have re-arranged the flow diagram to provide a more clear and detailed account of exclusions at each stage, including numbers and rationale for exclusion where appropriate. See the changes to Figure 1.

3. There are also places where the scope of the review needs tighter presentation. The review is framed around carbapenemase-producing Enterobacterales, but some included summaries and table entries still blur that focus by incorporating broader non-Enterobacterales material from regional review articles. This should be revised so that the evidence base and presentation remain fully consistent with the stated review question.

We have clarified further that studies that included organisms outside the scope were included here only if they also reported on Enterobacterales. We believe this is appropriate and aligned to the research question. We have also removed these non-Enterobacterales from Table 1.

*‘Studies reporting relevant Enterobacterales spp in addition to non-Enterobacterales spp (e.g., Acinetobacter baumannii or Pseudomonas aeruginosa) were eligible. However, in these cases, only those organisms within the scope (CPE) were part of the analysis. Studies that focused solely on non-Enterobacterales organisms were excluded, as were those conducted outside the Caribbean, even if CPE was described [Lines 182-187].’*

4. Finally, while some reviewer concerns were addressed substantively, others were addressed more by explanation than by revision. In particular, the concern that the Results still read too much like a listing of study findings rather than a more integrated narrative synthesis has not yet been fully resolved. For these reasons, I am inviting a further revision rather than rejecting the manuscript at this stage. Please submit a carefully revised version that resolves the methodological inconsistency around eligible study types, corrects the PRISMA accounting, tightens the scope of included evidence, and thoroughly cleans the manuscript for clarity and consistency throughout.

Thank you for the opportunity to further revise our manuscript and improve the Results section and the manuscript as requested above. We revisited the original reviewer comment to which this concern relates, namely that “the structure of narration just reported a list of the literature findings, without a clear discussion on the impact of such findings.” In our previous response, we explained that the manuscript structure was intentionally aligned with established scoping review approaches, whereby findings are mapped thematically within the Results section, supported by a summary table, with broader interpretation and discussion of impact synthesised within the Discussion section. In response to the editor’s further observations, we have undertaken additional revisions to strengthen the narrative synthesis throughout the manuscript and ensure that overarching trends and implications are more clearly conveyed. Specifically, we have clarified the analytical approach in the Methods section, revised the introductory framing of the Results section, and further integrated interpretation of common themes, emerging epidemiological patterns, and evidence gaps throughout the Results and Discussion sections.

The approach is stated in the methods, *‘A descriptive thematic analysis was applied to characterise and map the CPE epidemiology data from the identified studies [Lines 193-194].’*

The Results section has also been reorganised to include a short introduction that indicates the approach and cites the Table, before moving to the subheadings under which the data are mapped to the themes, *‘For each study, a summary of the CPE epidemiological data and key observations to support thematic analysis are presented in Table 1 [Lines 204-206]’*

Each subheading section has also been reviewed and changes made where appropriate to convey overall trends common to the studies and where a knowledge gap is notable. Similarly, the Discussion has been revised in several places with further holistic interpretation of the thematic analysis in the context of the wider literature in this area.

We trust that the revisions satisfactorily address your comments and concerns, and we are grateful for the opportunity to refine our manuscript further.

Yours sincerely,

Dr Saied Ali

On behalf of all authors
