# Supplementary material for: The Caribbean under the radar? A scoping review of carbapenemase-producing Enterobacterales across the region
Source: PLOS Glob Public Health. 2026 Aug 26;6(8):e0007176. doi: 10.1371/journal.pgph.0007176 (PMC13513946; doi:10.1371/journal.pgph.0007176)
Supplement: Peer review history

# Peer review: Decision Letter 0

**DOI:** 10.1371/journal.pgph.0007176.r001

**Authors:** Johanna Pruller

7 Jan 2026

PGPH-D-25-03527

The Caribbean under the radar? A scoping review of carbapenemase-producing Enterobacterales across the region

PLOS Global Public Health

Dear Dr. Ali,

Thank you for submitting your manuscript to PLOS Global Public Health. After careful consideration, we feel that it has merit but does not fully meet PLOS Global Public Health’s publication criteria as it currently stands. Therefore, we invite you to submit a revised version of the manuscript that addresses the points raised during the review process.

==============================

The manuscript has been evaluated by three reviewers, and their comments are available below.

The reviewers have raised a number of concerns that need attention. Could you please revise the manuscript to carefully address the concerns raised?

==============================

Please submit your revised manuscript by Feb 21 2026 11:59PM. If you will need more time than this to complete your revisions, please reply to this message or contact the journal office at globalpubhealth@plos.org. When you're ready to submit your revision, log on to https://www.editorialmanager.com/pgph/ and select the 'Submissions Needing Revision' folder to locate your manuscript file.

Please include the following items when submitting your revised manuscript:

- A letter that responds to each point raised by the editor and reviewer(s). You should upload this letter as a separate file labeled 'Response to Reviewers'.
- A marked-up copy of your manuscript that highlights changes made to the original version. You should upload this as a separate file labeled 'Revised Manuscript with Track Changes'.
- An unmarked version of your revised paper without tracked changes. You should upload this as a separate file labeled 'Manuscript'.

Guidelines for resubmitting your figure files are available below the reviewer comments at the end of this letter.

We look forward to receiving your revised manuscript.

Kind regards,

Johanna Pruller, Ph.D.

PLOS Staff Editor

PLOS Global Public Health

Journal Requirements:

1. Please amend your detailed Financial Disclosure statement. This is published with the article. It must therefore be completed in full sentences and contain the exact wording you wish to be published.

i. Please clarify all sources of financial support for your study. List the grants, grant numbers, and organizations that funded your study, including funding received from your institution. Please note that suppliers of material support, including research materials, should be recognized in the Acknowledgements section rather than in the Financial Disclosure.

ii. State the initials, alongside each funding source, of each author to receive each grant. For example: "This work was supported by the National Institutes of Health (####### to AM; ###### to CJ) and the National Science Foundation (###### to AM)."

iii. State what role the funders took in the study. If the funders had no role in your study, please state: “The funders had no role in study design, data collection and analysis, decision to publish, or preparation of the manuscript.”

iv. If any authors received a salary from any of your funders, please state which authors and which funders.

2. Please upload separate figure files in .tif or .eps format. Also, remove the figures from your manuscript file but keep the legends.

For more information about figure files please see our guidelines:

https://journals.plos.org/globalpublichealth/s/figures

https://journals.plos.org/globalpublichealth/s/figures#loc-file-requirements

3. We have noticed that you have uploaded Supporting Information files, but you have not included a list of legends. Please add a full list of legends for your Supporting Information files after the references list.

4. We note that your Data Availability Statement is currently as follows: All data underlying the results are fully available in the manuscript and no additional datasets were generated or required.

Please confirm at this time whether or not your submission contains all raw data required to replicate the results of your study. Authors must share the “minimal data set” for their submission. PLOS defines the minimal data set to consist of the data required to replicate all study findings reported in the article, as well as related metadata and methods (https://journals.plos.org/plosone/s/data-availability#loc-minimal-data-set-definition).

For example, authors should submit the following data:

- The values behind the means, standard deviations and other measures reported;

- The values used to build graphs;

- The points extracted from images for analysis.

Authors do not need to submit their entire data set if only a portion of the data was used in the reported study.

If your submission does not contain these data, please either upload them as Supporting Information files or deposit them to a stable, public repository and provide us with the relevant URLs, DOIs, or accession numbers. For a list of recommended repositories, please see https://journals.plos.org/plosone/s/recommended-repositories.

If there are ethical or legal restrictions on sharing a de-identified data set, please explain them in detail (e.g., data contain potentially sensitive information, data are owned by a third-party organization, etc.) and who has imposed them (e.g., an ethics committee). Please also provide contact information for a data access committee, ethics committee, or other institutional body to which data requests may be sent. If data are owned by a third party, please indicate how others may request data access.

If the reviewer comments include a recommendation to cite specific previously published works, please review and evaluate these publications to determine whether they are relevant and should be cited. There is no requirement to cite these works unless the editor has indicated otherwise.

Additional Editor Comments (if provided):

[Note: HTML markup is below. Please do not edit.]

Reviewers' comments:

Reviewer's Responses to Questions

**Comments to the Author**

1. Does this manuscript meet PLOS Global Public Health’s publication criteria?

Reviewer #1: Yes

Reviewer #2: Yes

Reviewer #3: Yes

**********

2. Has the statistical analysis been performed appropriately and rigorously?

Reviewer #1: N/A

Reviewer #2: N/A

Reviewer #3: N/A

**********

3. Have the authors made all data underlying the findings in their manuscript fully available (please refer to the Data Availability Statement at the start of the manuscript PDF file)?

The PLOS Data policy

Reviewer #1: Yes

Reviewer #2: Yes

Reviewer #3: Yes

**********

4. Is the manuscript presented in an intelligible fashion and written in standard English?

Reviewer #1: Yes

Reviewer #2: Yes

Reviewer #3: Yes

**********

Reviewer #1: This is a relevant and timely topic, and the authors successfully compile and summarize current information on CPE in the Caribbean. The manuscript is generally well written, with a clear structure and a methodology appropriate for a review article. The abstract and introduction provide sufficient context and highlight the importance of the subject within public health.

However, Table 1 appears excessively detailed and could benefit from a more concise presentation. In particular, the “Key Observations” column contains extensive descriptive text that could be reduced to the essential points. In the “Number of CPE Isolates” column, it would also be helpful to include, when available, the total number of isolates tested in each study, as this would improve comparability across the included works. Additionally, when the table lists “various” under “Detection & Diagnostic Methods,” it would be important to specify whether these methods are molecular or phenotypic. This distinction directly affects detection sensitivity and reliability, since a carbapenem-resistant phenotype does not necessarily indicate carbapenemase production, and conversely, some carbapenemase producers (e.g., OXA-48-like enzymes) may appear susceptible in vitro while still being clinically relevant.

Another aspect that requires clarification is the type of samples included in each study—whether environmental, human (clinical or colonization), or animal. Although the text briefly mentions that one study is environmental and others clinical, this information is not consistently reflected in Table 1 nor fully discussed in the manuscript. Explicitly identifying the source of the isolates is essential for understanding transmission dynamics and epidemiological significance. Because this information is missing, the conclusion also does not address how carbapenemase-producing Enterobacterales are distributed across different reservoirs. Expanding the conclusion to discuss the implications of detecting these resistances in clinical, environmental, and potentially animal samples would strengthen the manuscript, particularly from a One Health perspective, given the interconnectedness of human, animal, and environmental health in the spread of antimicrobial resistance.

Overall, the manuscript has strong potential, and addressing the points above would further enhance its clarity.

Reviewer #2: The manuscript is reviewing in a standardised way the research published on CPE in the Caribbean area. From the work published ii appears as a geographic area that is not sufficiently in focus to assess properly the level of antimicrobial resistance in Enterobacterales. Hopefully this manuscript will draw attention to researchers in the area to publish more information on AMR in this geographic region.

Reviewer #3: The authors reported a literature review on the occurence of carbapenemases in Caribean area. The study offer an interesting prospective of the carbapenem resistance burden but the results are not properly narrated. The structure of narration just reported a list of the literature findings, without a clear discussion on the impact of such findings. moreover, to better understand the impact of such circulation, information regarding plasmids types should be included. i suggest a re-evaluation of the type of narration and presentation of literature findings.

**********

what does this mean?). If published, this will include your full peer review and any attached files.

**Do you want your identity to be public for this peer review?**  If you choose “no”, your identity will remain anonymous but your review may still be made public.

For information about this choice, including consent withdrawal, please see our Privacy Policy

Reviewer #1: **Yes:**  Juliana Menezes

Reviewer #2: No

Reviewer #3: No

**********

[NOTE: If reviewer comments were submitted as an attachment file, they will be attached to this email and accessible via the submission site. Please log into your account, locate the manuscript record, and check for the action link "View Attachments". If this link does not appear, there are no attachment files.]

# Author comment: Author response to Decision Letter 1

**DOI:** 10.1371/journal.pgph.0007176.r002

26 Jan 2026

We thank the Editor and Reviewers for their constructive and thoughtful feedback. All comments have been carefully addressed and the manuscript has been revised accordingly.

Editorial requirements:

The Financial Disclosure statement has been fully revised to comply with journal wording requirements. Funding source, grant number (Health Research Board, ICAT-2022-001), award holder (SA), and the role of the funders are now clearly stated. We confirm the funders had no role in study design, data collection and analysis, decision to publish, or manuscript preparation, and that no author received salary support from the funders.

Figure files have been uploaded separately in the required format, removed from the manuscript text, and legends retained. A full Supporting Information legend list has been added after the references.

Regarding data availability, we confirm that all data underlying the findings are derived from published literature and are fully presented in the manuscript and Supporting Information (including Table 1, Figure 1, Results, and S1 File search strategies). No additional raw datasets were generated.

Reviewer 1:

Table 1 has been streamlined, particularly the “Key Observations” column. Where available, denominators have been added and the column heading revised to “Number of CPE Isolates / Total Number of Isolates Tested.” Diagnostic methods are now further clarified. Isolate source information has been clarified within the table. The Conclusion has been expanded to better contextualise findings across clinical and environmental reservoirs within a One Health framework.

Reviewer 2:

We appreciate the positive assessment. The manuscript aims to highlight the limited data from the Caribbean and encourage further regional AMR reporting.

Reviewer 3:

The Results follow a structured thematic synthesis in line with PRISMA-ScR guidance rather than listing individual studies. Broader interpretation is provided in the Discussion. Plasmid information has been included where reported in primary studies, and the lack of plasmid-level data across many studies has been discussed as an evidence gap.

We believe these revisions improve clarity, methodological transparency, and public health relevance, and we hope the manuscript is now suitable for further consideration.

Attachment Submitted filename: Response to Reviewers.docx

# Peer review: Decision Letter 1

**DOI:** 10.1371/journal.pgph.0007176.r003

**Authors:** Emma Campbell

16 Mar 2026

PGPH-D-25-03527R1

The Caribbean under the radar? A scoping review of carbapenemase-producing Enterobacterales across the region

PLOS Global Public Health

Dear Dr. Ali,

Thank you for submitting your manuscript to PLOS Global Public Health. After careful consideration, we feel that it has merit but does not fully meet PLOS Global Public Health’s publication criteria as it currently stands. Therefore, we invite you to submit a revised version of the manuscript that addresses the points raised during the review process.

Reviewer 4 has provided some further areas for improvement. Please review their comments and make the appropriate revisions.

Please submit your revised manuscript by Apr 27 2026 11:59PM. If you will need more time than this to complete your revisions, please reply to this message or contact the journal office at globalpubhealth@plos.org. When you're ready to submit your revision, log on to https://www.editorialmanager.com/pgph/ and select the 'Submissions Needing Revision' folder to locate your manuscript file.

Please include the following items when submitting your revised manuscript:

- A letter that responds to each point raised by the editor and reviewer(s). You should upload this letter as a separate file labeled 'Response to Reviewers'.
- A marked-up copy of your manuscript that highlights changes made to the original version. You should upload this as a separate file labeled 'Revised Manuscript with Track Changes'.
- An unmarked version of your revised paper without tracked changes. You should upload this as a separate file labeled 'Manuscript'.

Guidelines for resubmitting your figure files are available below the reviewer comments at the end of this letter.

We look forward to receiving your revised manuscript.

Kind regards,

Emma Campbell, Ph.D

Staff Editor

PLOS Global Public Health

Journal Requirements:

If the reviewer comments include a recommendation to cite specific previously published works, please review and evaluate these publications to determine whether they are relevant and should be cited. There is no requirement to cite these works unless the editor has indicated otherwise.

Additional Editor Comments (if provided):

[Note: HTML markup is below. Please do not edit.]

Reviewers' comments:

Reviewer's Responses to Questions

**Comments to the Author**

Reviewer #1: All comments have been addressed

Reviewer #3: All comments have been addressed

Reviewer #4: All comments have been addressed

**********

publication criteria?

Reviewer #1: Yes

Reviewer #3: Yes

Reviewer #4: Yes

**********

3. Has the statistical analysis been performed appropriately and rigorously?

Reviewer #1: N/A

Reviewer #3: N/A

Reviewer #4: Yes

**********

4. Have the authors made all data underlying the findings in their manuscript fully available (please refer to the Data Availability Statement at the start of the manuscript PDF file)?

The PLOS Data policy

Reviewer #1: Yes

Reviewer #3: Yes

Reviewer #4: Yes

**********

5. Is the manuscript presented in an intelligible fashion and written in standard English?

Reviewer #1: Yes

Reviewer #3: Yes

Reviewer #4: Yes

**********

Reviewer #1: The article is well written and well organized. The requested corrections have been properly addressed, and the authors have carefully considered and responded to the questions raised during the review process. The revisions have improved the clarity and overall quality of the manuscript. In its current form, the article is suitable for publication.

Reviewer #3: The comments were not fully followed and the narrative was not improved as requested.

Reviewer #4: 1. Definition of Caribbean and Its Implications

The geophysical definition is defensible but creates some anomalies worth acknowledging. Under this definition, Trinidad and Tobago and some mainland coastal territories may be included or excluded in ways that are not entirely transparent to readers unfamiliar with the region's geography. A brief supplementary note clarifying exactly which 20 nations/territories were included and which borderline cases were considered would improve.

Trinidad and Tobago — the only Caribbean country submitting data to WHO GLASS, and one with a relatively functional reference laboratory system — is entirely absent from the included studies despite being a logical candidate for CPE detection. The authors should acknowledge this paradox directly: GLASS participation does not guarantee published primary literature.

2.Inconsistency in Carbapenemase Nomenclature and Enzyme Classification

Throughout the manuscript, OXA-48 is referred to inconsistently — sometimes as "OXA-48" and elsewhere implicitly as "OXA-48-like." This distinction matters scientifically, as OXA-48-like enzymes include OXA-181, OXA-232, and others with differing hydrolytic profiles and epidemiological origins. The Dominican Republic environmental study detected OXA-72, which is an OXA-24/40-like enzyme (not OXA-48 producers as described at line 230-231:"OXA-48 producers were also reported in multi-country Caribbean studies and in environmental samples from the Dominican Republic").

3. Antimicrobial Stewardship and Regulatory Context

The discussion correctly identifies weak AMS frameworks and non-prescription antibiotic access as drivers of resistance, but the treatment is superficial. The Caribbean presents a highly fragmented regulatory landscape: some territories (UK Overseas Territories, French Departments like Guadeloupe and Martinique) operate under metropolitan European regulatory frameworks with prescription requirements, while independent nations vary enormously. This regulatory heterogeneity has direct consequences for selection pressure and CPE emergence, yet the authors treat the region as broadly homogeneous in this regard. Guadeloupe's regulatory environment, for instance, is materially different from Jamaica's or Haiti's, and this likely explains some of the epidemiological differences observed.

4. The PRISMA Flow Diagram Contains an Arithmetical Inconsistency

The flow diagram states 362 records identified but lists 137 (MEDLINE) + 636 (EMBASE) + 89 (Web of Science) = 862 records — not 362. This is a significant inconsistency that need to clarify. Does 362 records were screened from 862 records or otherwise?

**********

what does this mean?). If published, this will include your full peer review and any attached files.

**Do you want your identity to be public for this peer review?**  If you choose “no”, your identity will remain anonymous but your review may still be made public.

For information about this choice, including consent withdrawal, please see our Privacy Policy

Reviewer #1: **Yes:**  Juliana Menezes

Reviewer #3: No

Reviewer #4: No

**********

[NOTE: If reviewer comments were submitted as an attachment file, they will be attached to this email and accessible via the submission site. Please log into your account, locate the manuscript record, and check for the action link "View Attachments". If this link does not appear, there are no attachment files.]

# Author comment: Author response to Decision Letter 2

**DOI:** 10.1371/journal.pgph.0007176.r004

19 Mar 2026

Attachment Submitted filename: Response to Reviewers - Second Round.docx

# Peer review: Decision Letter 2

**DOI:** 10.1371/journal.pgph.0007176.r005

**Authors:** Bashar Haruna Gulumbe

5 Apr 2026

PGPH-D-25-03527R2

The Caribbean under the radar? A scoping review of carbapenemase-producing Enterobacterales across the region

PLOS Global Public Health

Dear Dr. Ali,

Thank you for submitting your manuscript to PLOS Global Public Health. After careful consideration, we feel that it has merit but does not fully meet PLOS Global Public Health’s publication criteria as it currently stands. Therefore, we invite you to submit a revised version of the manuscript that addresses the points raised during the review process.

Please submit your revised manuscript by May 20 2026 11:59PM. If you will need more time than this to complete your revisions, please reply to this message or contact the journal office at globalpubhealth@plos.org. When you're ready to submit your revision, log on to https://www.editorialmanager.com/pgph/ and select the 'Submissions Needing Revision' folder to locate your manuscript file.

Please include the following items when submitting your revised manuscript:

- A letter that responds to each point raised by the editor and reviewer(s). You should upload this letter as a separate file labeled 'Response to Reviewers'.
- A marked-up copy of your manuscript that highlights changes made to the original version. You should upload this as a separate file labeled 'Revised Manuscript with Track Changes'.
- An unmarked version of your revised paper without tracked changes. You should upload this as a separate file labeled 'Manuscript'.

Guidelines for resubmitting your figure files are available below the reviewer comments at the end of this letter.

As the corresponding author, your ORCID iD is verified in the submission system and will appear in the published article. PLOS supports the use of ORCID, and we encourage all coauthors to register for an ORCID iD and use it as well. Please encourage your coauthors to verify their ORCID iD within the submission system before final acceptance, as unverified ORCID iDs will not appear in the published article. *Only*  the individual author can complete the verification step; PLOS staff *cannot*  verify ORCID iDs on behalf of authors.

We look forward to receiving your revised manuscript.

Kind regards,

Bashar Haruna Gulumbe

Academic Editor

PLOS Global Public Health

Journal Requirements:

If the reviewer comments include a recommendation to cite specific previously published works, please review and evaluate these publications to determine whether they are relevant and should be cited. There is no requirement to cite these works unless the editor has indicated otherwise.

Additional Editor Comments (if provided):

The authors have made a clear effort to revise the manuscript, and the paper addresses an important and underrepresented public health issue with potential value. However, several issues still need to be resolved before it can be considered further.

1. The review methods and the included evidence are not yet fully aligned. The Methods state that only primary studies were eligible, but the included studies and Table 1 also contain narrative reviews and a systematic review/meta-analysis. This needs to be clarified and handled consistently THROUGHOUT the manuscript. I recommend that the authors review this table closely.

2. The study selection and PRISMA reporting also require further correction. Although the earlier EMBASE typo was explained, the flow diagram still does not read cleanly, particularly in how duplicates, screened records, full-text exclusions, and included studies are accounted for across stages. The numbers and exclusion categories should be presented in a way that is fully transparent and internally consistent.

3. There are also places where the scope of the review needs tighter presentation. The review is framed around carbapenemase-producing Enterobacterales, but some included summaries and table entries still blur that focus by incorporating broader non-Enterobacterales material from regional review articles. This should be revised so that the evidence base and presentation remain fully consistent with the stated review question.

4. Finally, while some reviewer concerns were addressed substantively, others were addressed more by explanation than by revision. In particular, the concern that the Results still read too much like a listing of study findings rather than a more integrated narrative synthesis has not yet been fully resolved.

For these reasons, I am inviting a further revision rather than rejecting the manuscript at this stage.

Please submit a carefully revised version that resolves the methodological inconsistency around eligible study types, corrects the PRISMA accounting, tightens the scope of included evidence, and thoroughly cleans the manuscript for clarity and consistency throughout.

[Note: HTML markup is below. Please do not edit.]

Reviewers' comments:

Reviewer's Responses to Questions

**Comments to the Author**

Reviewer #4: All comments have been addressed

**********

publication criteria?

Reviewer #4: Yes

**********

3. Has the statistical analysis been performed appropriately and rigorously?

Reviewer #4: Yes

**********

4. Have the authors made all data underlying the findings in their manuscript fully available (please refer to the Data Availability Statement at the start of the manuscript PDF file)?

The PLOS Data policy

Reviewer #4: Yes

**********

5. Is the manuscript presented in an intelligible fashion and written in standard English?

Reviewer #4: Yes

**********

Reviewer #4: (No Response)

**********

what does this mean?). If published, this will include your full peer review and any attached files.

**Do you want your identity to be public for this peer review?**  If you choose “no”, your identity will remain anonymous but your review may still be made public.

For information about this choice, including consent withdrawal, please see our Privacy Policy

Reviewer #4: **Yes:**  Truong Nhat My

**********

[NOTE: If reviewer comments were submitted as an attachment file, they will be attached to this email and accessible via the submission site. Please log into your account, locate the manuscript record, and check for the action link "View Attachments". If this link does not appear, there are no attachment files.]

# Author comment: Author response to Decision Letter 3

**DOI:** 10.1371/journal.pgph.0007176.r006

13 May 2026

Attachment Submitted filename: Response to Editor - Third Round.docx

# Peer review: Decision Letter 3

**DOI:** 10.1371/journal.pgph.0007176.r007

**Authors:** Sumanth Gandra

10 Aug 2026

The Caribbean under the radar? A scoping review of carbapenemase-producing Enterobacterales across the region

PGPH-D-25-03527R3

Dear Dr. Ali,

We are pleased to inform you that your manuscript 'The Caribbean under the radar? A scoping review of carbapenemase-producing Enterobacterales across the region' has been provisionally accepted for publication in PLOS Global Public Health.

Before your manuscript can be formally accepted you will need to complete some formatting changes, which you will receive in a follow up email. A member of our team will be in touch with a set of requests.

Please note that your manuscript will not be scheduled for publication until you have made the required changes, so a swift response is appreciated.

IMPORTANT: The editorial review process is now complete. PLOS will only permit corrections to spelling, formatting or significant scientific errors from this point onwards. Requests for major changes, or any which affect the scientific understanding of your work, will cause delays to the publication date of your manuscript.

If your institution or institutions have a press office, please notify them about your upcoming paper to help maximize its impact. If they'll be preparing press materials, please inform our press team as soon as possible -- no later than 48 hours after receiving the formal acceptance. Your manuscript will remain under strict press embargo until 2 pm Eastern Time on the date of publication. For more information, please contact globalpubhealth@plos.org.

Thank you again for supporting Open Access publishing; we are looking forward to publishing your work in PLOS Global Public Health.

Best regards,

Sumanth Gandra

Academic Editor

PLOS Global Public Health

***********************************************************

The authors adequately revised the manuscript as requested by previous reviewers.

Reviewer Comments (if any, and for reference):

Reviewer's Responses to Questions

**Comments to the Author**

Reviewer #4: All comments have been addressed

**********

publication criteria?

Reviewer #4: Yes

**********

3. Has the statistical analysis been performed appropriately and rigorously?

Reviewer #4: Yes

**********

4. Have the authors made all data underlying the findings in their manuscript fully available (please refer to the Data Availability Statement at the start of the manuscript PDF file)?

The PLOS Data policy

Reviewer #4: Yes

**********

5. Is the manuscript presented in an intelligible fashion and written in standard English?

Reviewer #4: Yes

**********

Reviewer #4: I have agreed to publish this manuscript in the past

**********

what does this mean?). If published, this will include your full peer review and any attached files.

**Do you want your identity to be public for this peer review?**  If you choose “no”, your identity will remain anonymous but your review may still be made public.

For information about this choice, including consent withdrawal, please see our Privacy Policy

Reviewer #4: No

**********
